# Correlates of individual voice and face preferential responses during resting state

**DOI:** 10.1038/s41598-022-11367-6

**Published:** 2022-05-03

**Authors:** Kathrin N. Eckstein, Dirk Wildgruber, Thomas Ethofer, Carolin Brück, Heike Jacob, Michael Erb, Benjamin Kreifelts

**Affiliations:** 1 Department of Psychiatry and Psychotherapy, Tübingen Center for Mental Health (TüCMH), University of Tübingen, Calwerstrasse 14, 72076 Tübingen, Germany; 2 Department for Biomedical Magnetic Resonance, University of Tübingen, Tübingen, Germany

**Keywords:** Neuroscience, Auditory system, Cognitive neuroscience, Sensory processing, Social neuroscience, Visual system

## Abstract

Human nonverbal social signals are transmitted to a large extent by vocal and facial cues. The prominent importance of these cues is reflected in specialized cerebral regions which preferentially respond to these stimuli, e.g. the temporal voice area (TVA) for human voices and the fusiform face area (FFA) for human faces. But it remained up to date unknown whether there are respective specializations during resting state, i.e. in the absence of any cues, and if so, whether these representations share neural substrates across sensory modalities. In the present study, resting state functional connectivity (RSFC) as well as voice- and face-preferential activations were analysed from functional magnetic resonance imaging (fMRI) data sets of 60 healthy individuals. Data analysis comprised seed-based analyses using the TVA and FFA as regions of interest (ROIs) as well as multi voxel pattern analyses (MVPA). Using the face- and voice-preferential responses of the FFA and TVA as regressors, we identified several correlating clusters during resting state spread across frontal, temporal, parietal and occipital regions. Using these regions as seeds, characteristic and distinct network patterns were apparent with a predominantly convergent pattern for the bilateral TVAs whereas a largely divergent pattern was observed for the bilateral FFAs. One region in the anterior medial frontal cortex displayed a maximum of supramodal convergence of informative connectivity patterns reflecting voice- and face-preferential responses of both TVAs and the right FFA, pointing to shared neural resources in supramodal voice and face processing. The association of individual voice- and face-preferential neural activity with resting state connectivity patterns may support the perspective of a network function of the brain beyond an activation of specialized regions.

## Introduction

Voices and faces are among the most salient cues in human life. This is reflected in the existence of specialized cerebral modules which are hierarchically organized and specifically tuned to respond to these cues. Core components for the primary identification of human voices and faces are the temporal voice area (TVA) for voices^1–4^ and the fusiform face area (FFA) for faces^5–8^. While not exclusively activated by these signals, they exhibit clearly voice- and face-preferential responses, respectively. The FFA together with the occipital face area (OFA) respond mainly to invariant facial features (e.g. gender)^5,8^. Further processing of dynamic face aspects, and integration of signals from voices and faces involves the posterior superior temporal sulcus (pSTS) and the thalamus^9–12^. The emotional information often present in faces and voices (e.g. in facial expressions and emotional prosody) additionally converges in the amygdala^9,13^. Further processing of such emotional information involves further regions such as the inferior frontal cortex (IFC) and orbitofrontal cortex (OFC)^14,15^. Convergent with the particular importance of voices and faces in human social communication, recent studies indicated that the responsivity to the preferred cues of the basic modules for identification of human voices and faces is moderated by interindividual differences in social signal processing, e.g. social anxiety^16^, and emotional intelligence^17^, even in the absence of emotional information. In some cases, as described above e.g. for the pSTS and thalamus, the hemodynamic correlates of cerebral processing of signals from different sensory modalities overlap. This phenomenon will be termed supramodal throughout this manuscript.

While a plethora of neuroimaging studies delineated the neural networks that are active when we see faces or hear voices, it remains a completely open question if the brain’s activity patterns also reflect the individual cerebral responsivity to voices and faces in the absence of these cues and if these representations may share neural substrates across sensory modalities.

During the past three decades, the resting brain has become a major research focus as it became clear that spontaneous physiological low-frequency fluctuations in brain activity occur non-randomly but simultaneously in various, partially overlapping neural networks in the absence of any cues or stimulation or cognitive/emotional task^18^. Nevertheless, these fluctuation patterns are not independent from individual traits or diseases, as they have been shown to correlate with various aspects of behavioural tendencies^19–21^, personality^22,23^, psychopathology^21,24,25^, and psychiatric disease (e.g. dementia and schizophrenia^26^) also demonstrating that resting state data can be used to expand the neuroimaging perspective on their cerebral representation in a complementary manner with the potential to detect links between the neural networks underlying various perceptual, cognitive or emotional functions not apparent in stimulation-based designs.

In the area of face and voice processing, correlations of resting state functional connectivity (RSFC) with behavioural outcomes, e.g. performance in various face- and voice-processing tasks have been observed^27–30^. One study compared functional connectivity patterns during resting state and a passive viewing task and found for both conditions similar networks including posterior fusiform gyrus, inferior occipital gyrus and superior temporal sulcus^27^. In this work the informative RSFC patterns were found exclusively within the network of modality-specific preferential processing areas^27^. Two studies combined RSFC in the face processing network with behavioural performance in a face identification task and an emotional face matching task, respectively^28,29^ and found RSFC patterns between modality-specific preferential processing areas but also with other parts of the brain^28,29^. One study in children revealed that performance in an auditory emotional prosody recognition task was predicted by stronger connectivity between the inferior frontal gyrus and motor regions. Here, informative RSFC patterns were found exclusively outside the modality-specific preferential processing networks^30^.

In the present study, we intended to determine the neural correlates of voice- and face-preferential responses in the absence of voices and faces in the resting state. Furthermore, we aimed to identify brain areas with RSFC patterns supramodally reflecting preferential responses to both, voices and faces. To this end, 60 healthy individuals underwent functional magnetic resonance imaging (fMRI) at rest and during stimulation with voices, faces and various other classes of acoustic and visual stimuli. Individual voice- and face-preferential responses were correlated with RSFC employing multi voxel pattern analyses (MVPA) and seed-based analyses focused on TVA and FFA.

## Materials and methods

### Participants

60 healthy individuals (mean age 25.8 years, s.d. = 4.5 years, 30 female) participated at the University of Tübingen. All of the participants were native German speakers and right-handed, as assessed with the Edinburgh Inventory^31^. None of the participants was taking any regular medication, or had a history of substance abuse, or psychiatric or neurological illness. Hearing was normal, vision normal or corrected to normal in all participants. The study was performed according to the Code of Ethics of the World Medical Association (Declaration of Helsinki) and the protocol of human investigation was approved by the local ethics committees where the study was performed (i.e., the medical faculties of the Universities of Tübingen and Greifswald). All individuals gave their written informed consent prior to their participation in the study.

### Stimuli and experimental design

Two fMRI experiments were performed to localize face-sensitive^5^ and voice-sensitive^1^ brain areas as described in previous publications^9,10,14,16,17,32,33^: For the face-sensitivity experiment, pictures from four different categories (faces, houses, objects, and natural scenes) were employed within a block design. All stimuli used in the experiment were black-and-white photographs unknown to the participants^17^. The shown face stimuli had no obvious emotional connotation, but rather showed neutral facial expressions. The house stimuli were multilevel apartment houses from different materials (brick, wooden, concrete). As object stimuli different everyday life items were used (e.g. flat iron, spoon, T-shirt). The fourth category of natural scenes represented different countryside pictures (e.g. mountain, coastline, waterfall). Each block and category contained 20 stimuli^17^. Within blocks, the stimuli were presented in random order for 300 ms. Stimuli were separated by 500 ms periods of fixation [1 block = 20 stimuli × (300 ms picture + 500 ms fixation) = 16 s]. Eight blocks of each category pseudorandomized within the experiment were shown separated by short ~ 1.5 s rest periods^17^. A one-back task was employed, in which the participants had to press a button on a fibre optic system (LumiTouch, Photon Control, Burnaby, Canada) with their right index finger when they saw a picture twice in a row, to ascertain constant attention^17^. The appearance of repeated stimuli was pseudorandomized ensuring a distribution across the entire experiment. Visual stimuli were back-projected onto a screen placed in the magnet bore behind the participant’s head and viewed by the participant through a mirror system mounted onto the head coil.

The voice-sensitivity experiment was developed based on the study by Belin et al.^1^ in form of a block design experiment with 24 stimulation blocks and 12 silent periods (each 8 s) in a passive-listening design without an explicit task. Between the blocks were short periods without sound (2 s). Participants were instructed to listen attentively with their eyes closed. The stimulus material comprised 12 blocks of human vocal sounds (speech, sighs, laughs, cries), 6 blocks of animal sounds (e.g., gallops, various cries) and 6 blocks of environmental sounds (e.g., cars, planes, doors, telephones). Stimuli were normalized with respect to mean acoustic energy^17^. Sound and silence blocks were pseudorandomized across the experiment with the restriction that with the restriction that no two blocks of silence directly followed each other.

Both experimental designs have been validated in previous studies^9,10,14,17,32,33^. Further details on the stimulus material and experimental designs have been reported elsewhere^9^.

For the resting state measurements (duration about 7 min and 15 s), the participants were instructed to keep their eyes closed with no further task.

### Image acquisition

MRI data were acquired with a TRIO 3T and a PRISMA scanner (Siemens, Erlangen, Germany). Structural T1-weighted images (176 slices, TR = 2300 ms, TE = 2.96 ms, TI = 1100 ms, voxel size: 1 × 1 × 1 mm^3^) and functional images (30 axial slices captured in sequential descending order, 3 mm thickness + 1 mm gap, TR = 1.7 s, TE = 30 ms, voxel size: 3 × 3 × 4 mm^3^, field of view 192 × 192 mm^2^, 64 × 64 matrix, flip angle 90°) were recorded. For the resting state measurements, 245 images were recorded. The activation tasks were performed after completion of the resting state measurements to avoid carry-over effects. The time series comprised 368 images for the face experiment and 232 images for the voice experiment and 250 images for the resting state measurement. A field map with 36 slices (slice thickness 3 mm, TR = 400 ms, TE(1) = 5.19 ms, TE(2) = 7.65 ms) was recorded.

### Analysis of fMRI data

Statistical parametric mapping software (SPM8, Wellcome Department of Imaging Neuroscience, London, http://www.fil.ion.ucl.ac.uk/spm) was used to analyse the imaging data. Pre-processing generally included the removal of the first five EPI images from each run to exclude measurements preceding T1 equilibrium.

#### Face- and voice-sensitivity experiments

The preprocessing procedure consisted of realignment, unwarping using a static field map, coregistration of anatomical and functional images, segmentation of the anatomical images, normalization into MNI space (Montreal Neurological Institute^34^) with a resampled voxel size of 3 × 3 × 3 mm^3^, temporal smoothing with a high-pass filter (cut-off frequency of 1/128 Hz) and spatial smoothing employing a Gaussian kernel (8 mm full width at half maximum, FWHM). The response to the single categories (faces (F), houses (H), objects (O), and natural scenes (S) in the face localizer as well as vocal sounds (V), animal sounds (A), and environmental sounds (E) in the voice localizer were independently modelled with a box-car function corresponding to the duration of the stimulation blocks (16 s in the face localizer and 8 s in the voice localizer) convolved with the hemodynamic response function (HRF). The error term was calculated as a first order autoregressive process with a coefficient of 0.2 and a white noise component accounting for serial autocorrelations^35^. To minimize motion-associated error variance, the six motion parameters (i.e. translation and rotation on the x-, y-, and z-axes) were included in the single subject models as covariates.

Contrast images were constructed using data from the first-level general linear models [face-sensitivity: F > (H, O, S); voice-sensitivity: V > (A, E)] for each subject. Taking these contrast images as sources, a second-level random-effect analysis was performed with one-sample t-tests to define the face-sensitive fusiform face area (FFA) and the voice-sensitive temporal voice area (TVA) as functional regions of interest (ROI) for further analyses. Statistical significance of activations was assessed at p < 0.001, uncorrected at voxel level and with FWE correction for multiple comparisons at cluster level with p < 0.05. For the definition of the FFA, the fusiform gyrus was taken as a priori anatomical ROI; for definition of the TVA, the temporal gyri and the temporal pole were selected. For definition of the functional ROIs (i.e. FFA and TVA), FWE-cluster level correction was performed across these a priori anatomical ROIs using small volume correction (SVC^36^). We picked the maximum activation in the fusiform gyrus for the FFA and in the temporal lobe for the TVA respectively, and defined the surrounding 100 most sensitive voxels as masks for the functional ROIs. Within these ROIs individual voice- and face-preferential responses were assessed using minimum difference criteria (for voices V > max[A, E], for faces F > max[H, O, S])^37^. Intercorrelations of the four regressors were evaluated. Differences in the face- and voice-sensitive and -preferential responses between both hemispheres and interhemispheric differences in cue-sensitivity and -preferentiality between TVA and FFA were post hoc tested using two-sided paired t-tests with Bonferroni correction.

#### Resting state functional connectivity analysis

For RSFC analyses we used the CONN toolbox (v 16b^38^) implemented in SPM8. The spatial preprocessing was performed analogously to the procedure described for the face- and voice-sensitivity experiments. Denoising included linear regression of the following confounding effects: White matter and CSF components (6P each), effect of rest (2P, temporal component and first order derivates) and motion regression (12 regressors: 6 motion parameters and 6 first-order temporal derivates) and band-pass filtering (0.008–0.09 Hz). Linear detrending was added to remove linear trends.

The participants’ movement parameters, their first order derivatives and the BOLD signal from white matter, cerebrospinal fluid and effect of rest (each with five temporal components) were included in the analysis as covariates to reduce their confounding influences. In the individual first-level analyses, bivariate correlation coefficients were calculated as linear measures of functional connectivity for the ensuing analyses. Coefficients were Z transformed to achieve comparability for group-level analyses, and gender, age and scanner were included as regressors of no interest. The Automated Anatomic Labelling (AAL) toolbox^39^ was used for the definition of anatomical regions in MNI space. The analysis targeted the correlation of individual resting state functional connectivity (RSFC) with face-/voice-preferential responses both with defined regions of interest (ROIs) and at whole brain level. To this end, analyses were done on two different levels: ROI-to-voxel analyses should detect associations between individual voice- and face-preferential responses of the ROIs and their RSFC with other brain regions. Here, the significance of observed connectivity patterns was assessed using a threshold of p < 0.001 at voxel level, two-tailed with FWE correction (p < 0.05) for multiple comparisons at cluster level. Results were Bonferroni-corrected for the numbers of regressors (4) and ROIs (4), so that the effective cluster threshold amounted to p < 0.00315. Second, a spatial hypothesis-free strategy was implemented using voxel-to-voxel multivariate multi voxel pattern analyses (MVPA). Here, for each voxel separately, a low-dimensional multivariate representation of the connectivity pattern between this voxel and all other voxels in the brain was calculated. This representation was based on a principal component analysis of the inter-subject variability of each separate voxel’s connectivity pattern enabling the investigation of differences across subjects using second-level multivariate analyses. The number of principal components was set to three and number of dimensions was set to 64 (dimensionality reduction)^40^. The goal of the group-MVPA approach was to detect whole brain resting state functional connectivity patterns correlating with individual voice-preferential responses of the TVA (i.e., V > max[A,E]) and face-preferential responses (i.e., F > max[H,O,S]) of the FFA. These individual estimates were used as group level regressors in the RSFC analyses (four regressors: two for the FFAs, two for the TVAs). Results were evaluated at a voxel-wise threshold of p < 0.001 and whole brain FWE-corrected at cluster level with additional Bonferroni-correction for the number of tested regressors (4) resulting in an effective cluster threshold of p < 0.0125. Findings of the MVPA were further analysed using the significant clusters as seeds for ensuing seed-to-voxel analyses. Convergence of RSFC patterns between different seeds was tested using conjunction analyses with a minimum statistic^41^. Results were assessed at a voxel-wise threshold of p < 0.001 and whole brain FWE-corrected at cluster level with a cluster threshold of p < 0.05.

## Results

### ROI characteristics

**Figure 1:**
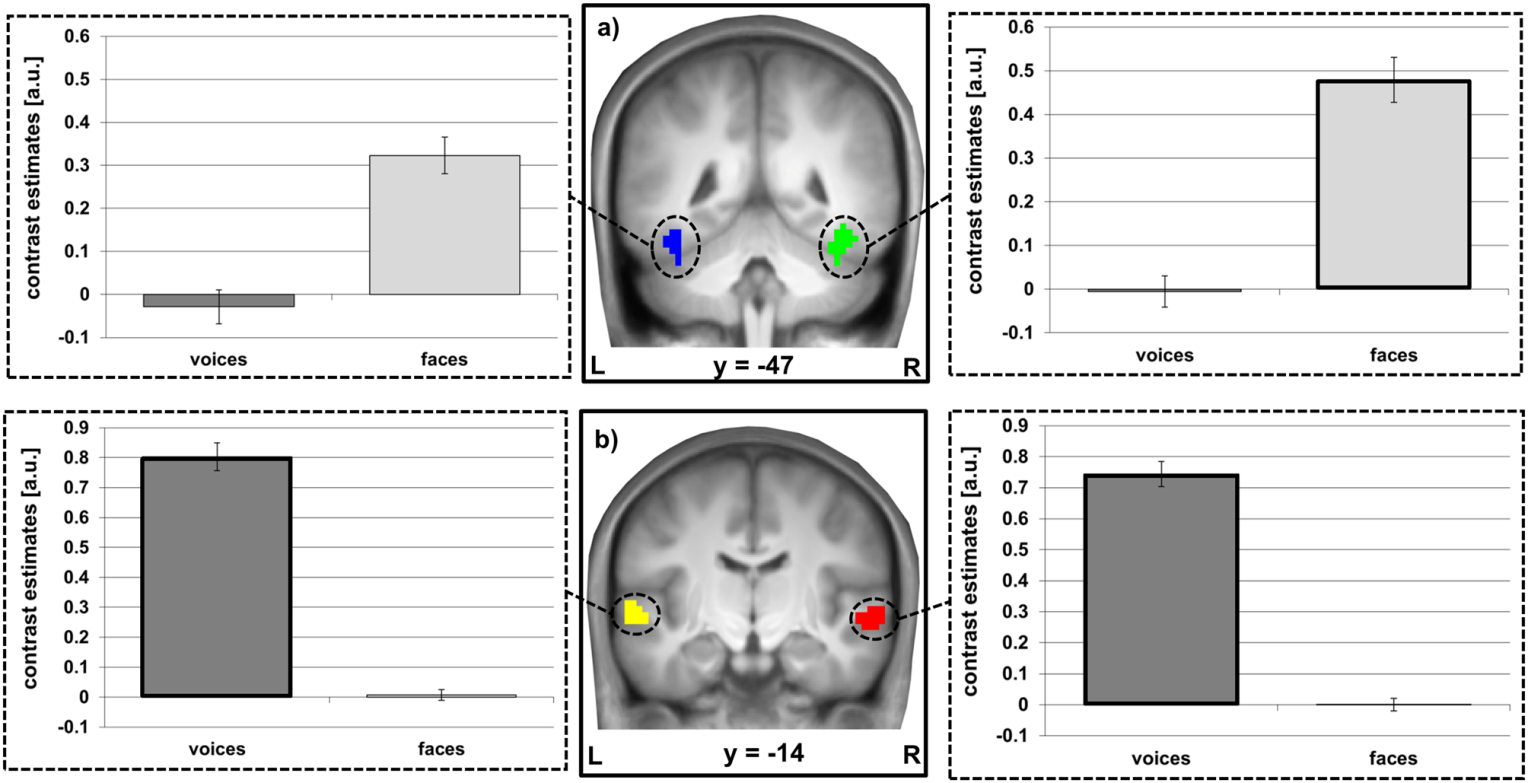
Face and voice processing areas. (**a**) The fusiform face area (rFFA in green, lFFA in blue), and (**b**) the temporal voice area (rTVA in red, lTVA in yellow), rendered onto the mean anatomical scan of the study population. The functional ROIs (i.e. FFA and TVA) were identified selecting the maximum activation in the fusiform gyrus and in the temporal lobe respectively, and defining the surrounding 100 most sensitive voxels as masks. Face-sensitivity is given as F > (H, O, S), voice-sensitivity as V > (A, E) for each subject. Individual voice- and face-preferential responses were assessed using minimum difference criteria (for voices V > max[A, E], for faces F > max[H, O, S]). The bars depict mean voice- and face-sensitivity of each region. Bold frames indicate that these regions respond also preferentially to the highlighted cues as compared to each of the experimental comparators (for details see “Materials and methods” section). Coordinates refer to MNI space. *R* right, *L* left. Error bars indicate the standard error of the mean. Additional material is available in the Supplement: Supplemental Fig. 1 depicts whole brain slices of the functional ROIs FFA and TVA.

The activation pattern of the right and left FFA showed a significant sensitivity for faces (rFFA t = 9.321, p < 0.001 and lFFA t = 7.585, p < 0.001), whereas significant face-preferential responses were observed in the right FFA (t = 4.344, p < 0.0001), but not the left FFA (t = 0.624, p = 0.535). The bilateral TVAs were highly sensitive to and preferential for voices (sensitivity: rTVA t = 18.265, p < 0.0001 and lTVA t = 17.457, p < 0.001; preferentiality: rTVA t = 14.456, p < 0.001 and lTVA t = 14.023, p < 0.001). ROI characteristics are graphically displayed in Fig. 1. The ROIs’ preferential responses to their preferred cues were significantly correlated within modality (voices: r(58) = 0.74, p < 0.001; faces: r(58) = 0.60, p < 0.001) but not across modalities (all abs(r(58)) < 0.12, all p > 0.05).

Comparison between the right and left hemisphere revealed no significant difference for voice-sensitivity or -preferentiality (all t < 2.03), all p > 0.187), but significant differences for face-sensitivity and -preferentiality in favour of the right hemisphere (all t > 3.75, all p < 0.004). Comparison of hemispheric differences in modality-specific differences in cue sensitivity and preferentiality between TVA and FFA corroborated the difference between the sensory modalities, both for sensitivity and for preferentiality (all t > 3.93, p < 0.002), i.e. a greater hemispheric difference in face-sensitivity and -preferentiality than in voice-sensitivity and -preferentiality.

### ROI-to-voxel analysis

In this analysis, only individual voice-preferential responses of the lTVA were significantly associated with RSFC between the lTVA and a cluster in the right supramarginal gyrus extending into the inferior parietal gyrus (peak: − 57x − 66y 27z; 143 voxels; p(FWE-corr.) = 0.0018).

### Multi-voxel pattern analysis (MVPA)

**Figure 2:**
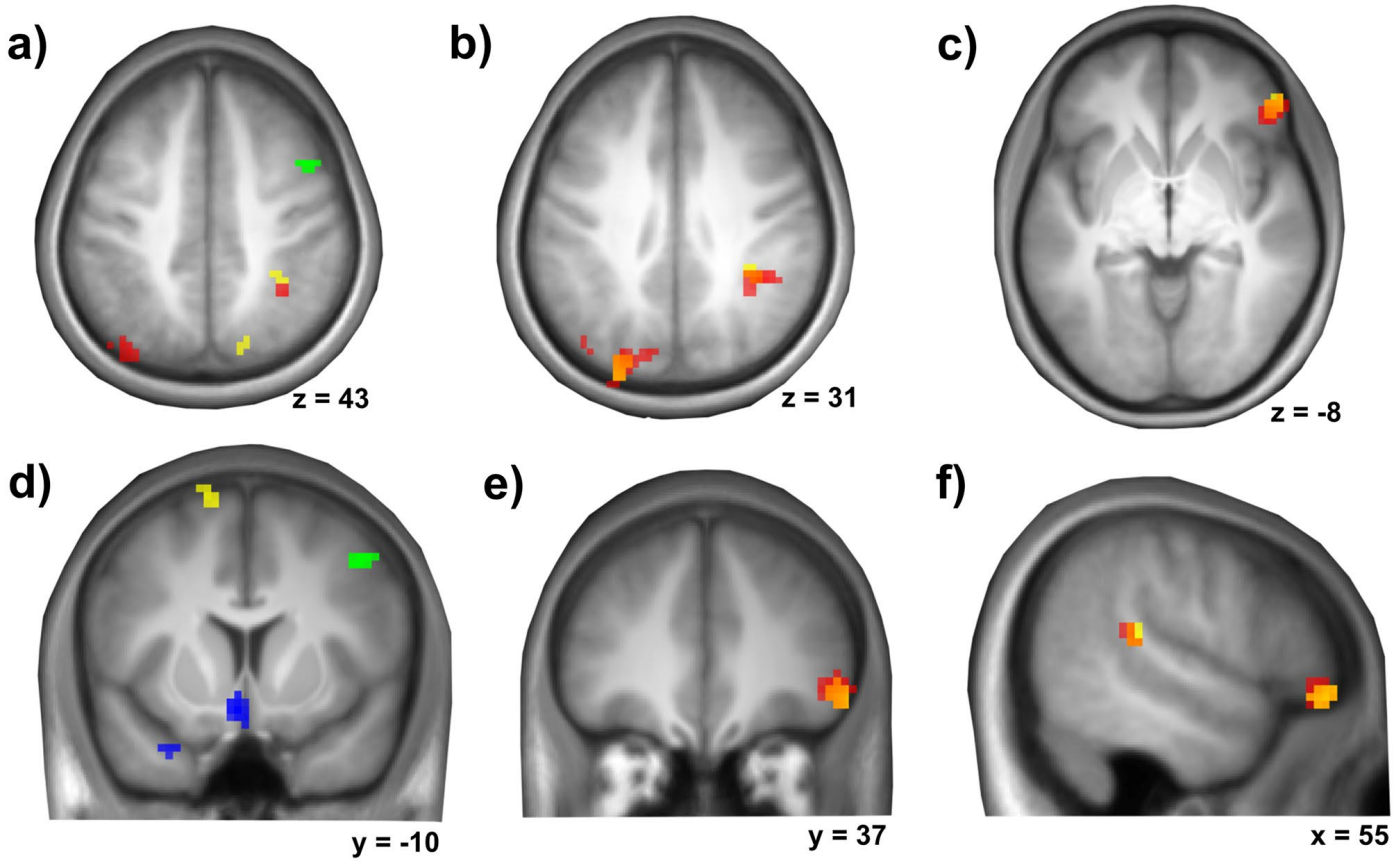
Multi-voxel pattern analyses: correlates of voice- and face-preferential responses. Areas of the RSFC patterns which significantly correlate with the individual responses to the preferred cues of FFA and TVA (red/yellow: right/left TVA voice-preferential responses (**a–f**); orange: overlap of right/left TVA voice-preferentiality correlates (**b,c,e,f**); green/blue: right/left FFA face-preferentiality correlates (**a,d**). Underlying RSFC patterns of informative clusters not shown here. Results are shown at a voxel-wise threshold of p < 0.001 and whole brain FWE-corrected at cluster level with additional Bonferroni-correction for the number of tested regressors (4) leading to an effective cluster threshold of p < 0.0125.

Using rFFA face-preferential responses as regressor, we identified one informative cluster in the right middle frontal gyrus extending into the precentral gyrus. For the lFFA two clusters in the left caudate nucleus/olfactory gyrus and left superior temporal pole were evident. For the rTVA and lTVA voice-preferential responses four overlapping clusters emerged: in the left superior occipital gyrus, the right inferior parietal gyrus, the right superior temporal gyrus and the right frontal inferior orbital gyrus. For rTVA voice-preferentiality two additional clusters were detected in the left middle occipital gyrus and the right thalamus, for the left TVA two additional clusters were located in the left frontal superior gyrus and the right parietal superior gyrus. A detailed description of the clusters can be found in Table 1. A graphical representation is displayed in Fig. 2.

**Table 1:** Multi-voxel pattern analysis (MVPA).

	Anatomical structure	Peak voxel	Cluster size (vx)	p
rFFA	R middle frontal gyrus/precentral gyrus	+ 45 + 09 + 45	14	0.00192
lFFA	L caudate nucleus and olfactory gyri	− 06 + 06 − 15	45	0.000036
	L superior temporal pole	− 27 + 06 − 30	15	0.001948
rTVA	**L superior occipital gyrus**	− 24 − 81 + 33	91	0.000036
	**R inferior parietal gyrus**	+ 36 − 51 + 42	49	0.000036
	**R superior temporal gyrus**	+ 54 − 36 + 15	13	0.006604
	**R frontal inferior orbital gyrus**	+ 48 + 36 − 06	50	0.000036
	L middle occipital gyrus	− 36 − 81 + 48	42	0.000036
	R thalamus	+ 06 − 21 + 21	14	0.003756
lTVA	**L superior occipital gyrus**	− 27 − 81 + 33	51	0.000036
	**R inferior parietal gyrus**	+ 36 − 42 + 36	27	0.00004
	**R superior temporal gyrus**	+ 57 − 33 + 15	24	0.00006
	**R frontal inferior orbital gyrus**	+ 51 + 39 − 09	26	0.00004
	L frontal superior gyrus	− 15 + 06 + 69	17	0.000716
	R parietal superior gyrus	+ 15 − 78 + 48	13	0.00622

RSFC correlates of individual voice- and face-preferential responses in TVA and FFA.

Voxel-wise threshold was set to p < 0.001 and whole brain FWE-corrected at cluster level with additional Bonferroni-correction for the number of tested regressors (4) leading to an effective cluster threshold of p < 0.0125.

*R* right, *L* left.

Overlapping clusters for the rTVA and lTVA are marked in bold. Voxel size 3 × 3 × 4 mm^3^.

For the four overlapping clusters informative of both the rTVA and lTVA voice-preferential responses common regions were calculated and further on used as seeds. The characteristics of the resulting clusters are described in Table 2.

**Table 2:** Multi-voxel pattern analysis (MVPA).

	Anatomical structure	Peak voxel	Cluster size (vx)	p
rlTVA	L superior occipital gyrus	− 27 − 81 + 33	42	0.000009
	R inferior parietal gyrus	+ 36 − 45 + 42	14	0.000939
	R superior temporal gyrus	+ 54 − 36 + 15	9	0.019070
	R frontal inferior orbital gyrus	+ 51 + 39 − 09	23	0.000019

Overlapping clusters informative of both the rTVA and lTVA voice-preferential responses.

Voxel-wise threshold was set to p < 0.001 and whole brain FWE-corrected at cluster level with a cluster threshold of p < 0.05. Voxel size 3 × 3 × 4 mm^3^.

*R* right, *L* left.

Significant clusters were used as seeds for subsequent post-hoc explanatory seed-to-voxel analyses.

**Figure 3:**
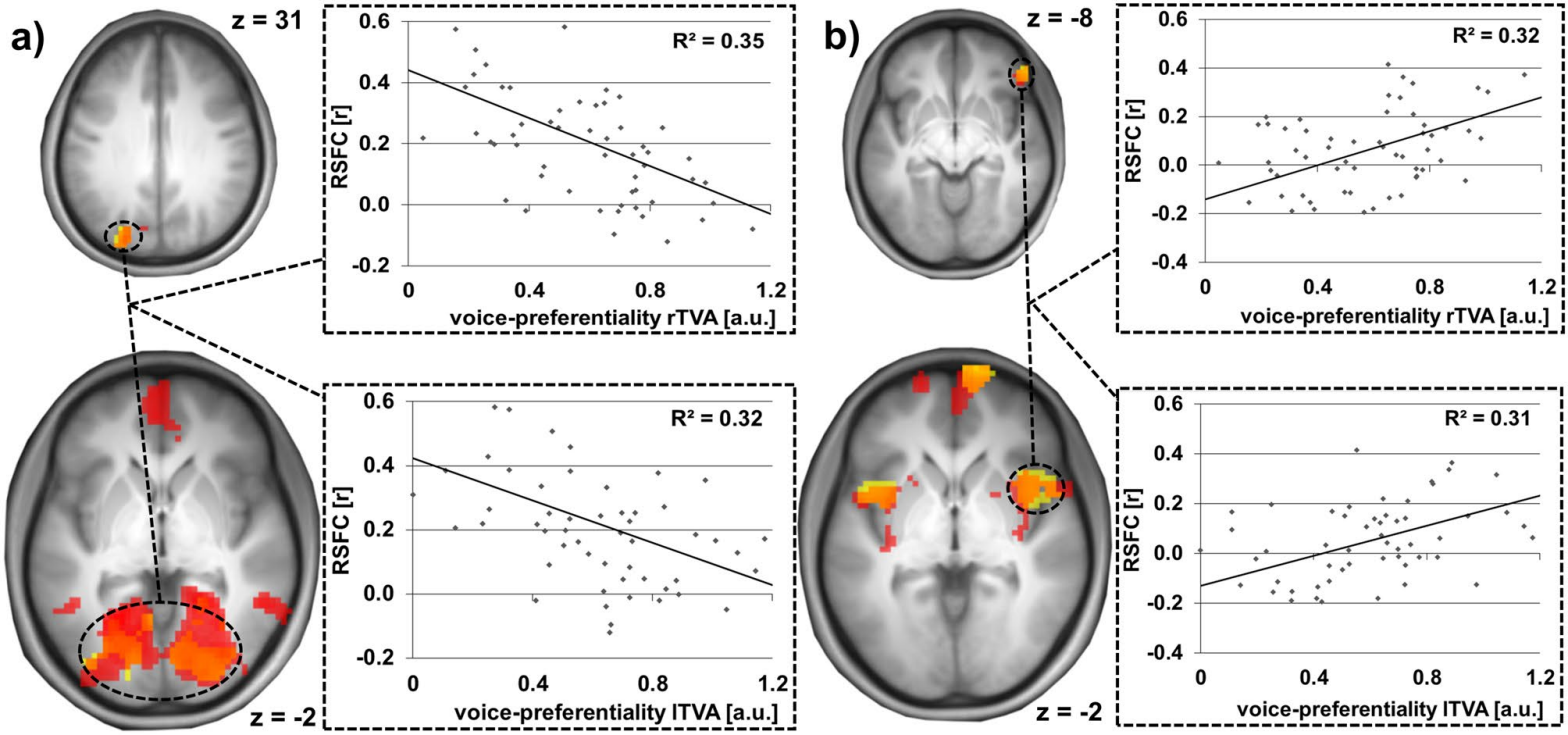
Convergence of informative RSFC patterns for bilateral TVA voice-preferentiality using (**a**) the left superior occipital gyrus and (**b**) the right inferior orbital gyrus as seed region. Top brain section: Exemplary MVPA cluster informative of right and left TVA voice-preferentiality used as seed region [see also Fig. 2b,c]. Bottom brain section: The seed’s convergent informative RSFC patterns regarding individual voice-preferential responses of the right (red) and left (yellow) TVA (orange: overlap of informative patterns for right and left TVA voice-preferentiality) as evaluated post hoc. Results shown at a voxel-wise threshold of p < 0.001 and whole brain FWE-corrected at cluster level with a cluster threshold of p < 0.05. Coordinates refer to MNI space. The diagrams on the right illustrate the underlying association of voice-preferential responses and RSFC.

For the TVAs the convergence of informative MVPA clusters was accompanied by a relatively strong convergence of their RSFC patterns in contrast to the FFAs’ RSFC patterns. Tables 3, 4 and 5 give an overview of convergent RSFC clusters across all informative regions observed in the MVPA analysis. Convergent clusters for the bilateral TVAs are listed in Table 3, exemplary graphical representations are given in Fig. 3.

**Table 3:** Convergent RSFC patterns informative of bilateral TVA voice-preferential responses.

Seed cluster	Direction of correlation	Anatomical structure	Peak voxel	Cluster size (vx)	p
L superior occipital gyrus (Fig. 3a)	Negative	R and L calcarine cortex/R and L lingual gyri/R and L cuneus/R and L superior, middle and inferior occipital gyri	36 − 63 − 9	1311	< 0.001
	Positive	L middle frontal gyrus/L inferior frontal gyrus, pars triangularis	− 42 36 36	75	0.017
R inferior parietal gyrus	Negative	R precuneus	21 − 54 27	79	0.011
R superior temporal gyrus		No suprathreshold cluster			
R frontal inferior orbital gyrus (Fig. 3b)	Positive	R and L cingulum/R and L supplementary motor area	− 6 3 42	393	< 0.001
	Positive	R insula/R Rolandic operculum/R frontal inferior operculum/R putamen R superior temporal pole	27 21 3	211	< 0.001
	Positive	R supramarginal gyrus/R superior temporal lobe/R postcentral lobe	69 − 24 24	187	< 0.001
	Positive	L Insula/L Rolandic operculum/L frontal inferior operculum/L superior temporal pole	− 36 3 3	109	0.002
	Positive	L superior parietal gyrus/L precuneus	− 15 − 48 57	60	0.031
	Negative	R medial orbital gyrus/R superior frontal gyrus, medial/R superior frontal gyrus, orbital	12 72 − 3	89	0.006
	Negative	R and L cerebellum lobule IX	3 − 54 − 45	55	0.042

Results are shown at a voxel-wise threshold of p < 0.001 and whole brain FWE-corrected at cluster level with a cluster threshold of p < 0.05. Voxel size 3 × 3 × 4 mm^3^.

*R* right, *L* left.

**Table 4:** Number of intramodal convergent RSFC clusters using the MVPA clusters with the RSFC correlates of individual voice-preferential responses in the right and left TVA.

MVPA cluster	rTVA L sup occ	rTVA R inf par	rTVA R sup temp	rTVA R front inf	rTVA L mid occ − 36 − 81 48	rTVA R thalamus 6 − 21 21	lTVA L sup occ	lTVA R inf par	lTVA R sup temp	lTVA R front inf	lTVA L front sup − 15 6 69
rTVA L sup occ											
rTVA R inf par	–										
rTVA R sup temp	–	–									
rTVA R front inf	–	–	*1*								
rTVA L mid occ − 36 − 81 48	*2*	–	–	–							
rTVA R thalamus 6 − 21 21	–	–	–	–	–						
lTVA L sup occ	**2**	–	–	–	–	–					
lTVA R inf par	–	**1**	–	–	–	–	–				
lTVA R sup temp	–	–	–	–	–	–	–	–			
lTVA R front inf	–	–	–	**7**	–	–	–	–	–		
lTVA L front sup − 15 6 69	*2*	–	–	*2*	–	–	–	–	–	*2*	
lTVA R pariet sup 15 − 78 48	*1*	–	–	*1*	–	–	*1*	–	–	–	–

Results are shown at a voxel-wise threshold of p < 0.001 and whole brain FWE-corrected at cluster level with a cluster threshold of p < 0.05.

*R* right, *L* left.

Voice-preferentiality is encoded in bold and italics. The bold signifies significant clusters between the corresponding right and left TVA region, italics between different TVA regions. Voxel size 3 × 3 × 4 mm^3^.

**Table 5:** Number of supramodal convergent RSFC clusters using MVPA clusters with the RSFC correlates of individual face-preferential responses in the right and left FFA with RSFC correlates of individual voice-preferential responses in the right and left TVA.

MVPA cluster	rTVA L sup occ	rTVA R inf par	rTVA R sup temp	rTVA R front inf	rTVA L mid occ − 36 − 81 48	rTVA R thalamus 6 − 21 21	lTVA L sup occ	lTVA R inf par	lTVA R sup temp	lTVA R front inf	lTVA L front sup − 15 6 69	lTVA R pariet sup 15 − 78 48
rFFA R mid front	**4**	–	–	**1**	–	–	–	–	–	–	**1**	–
lFFA L caudate − 6 6 − 15	**1**	–	–	–	–	–	**1**	–	–	–	–	–
lFFA L sup temp − 27 6 − 30	–	–	–	–	–	–	–	–	–	–	–	–

Results are shown at a voxel-wise threshold of p < 0.001 and whole brain FWE-corrected at cluster level with a cluster threshold of p < 0.05.

*R* right, *L* left.

Supramodal conjunctions are encoded in bold. Voxel size 3 × 3 × 4 mm^3^.

**Figure 4:**
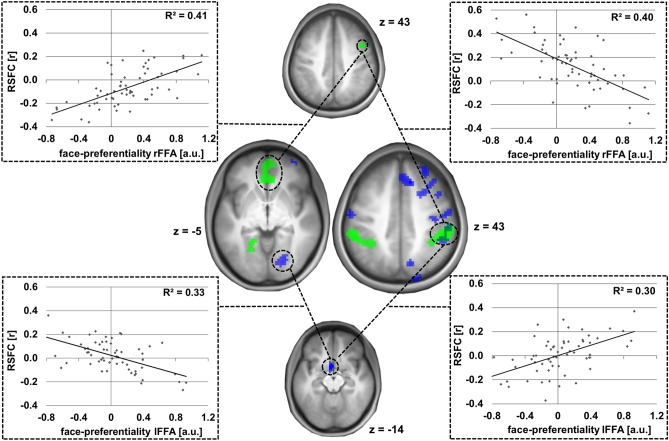
Divergence of informative RSFC patterns for bilateral FFA face-preferentiality. Individual face-preferential responses were assessed using minimum difference criteria (F > max[H, O, S]). Top and bottom middle: Two exemplary MVPA clusters informative of right (green) and left (blue) FFA face-preferentiality used as seed regions [see also Fig. 2b]. Centre middle: The seeds’ RSFC patterns associated with individual right (green) and left (blue) FFA face-preferentiality as evaluated post hoc. Results shown at a voxel-wise threshold of p < 0.001 and whole brain FWE-corrected at cluster level with a cluster threshold of p < 0.05. Coordinates refer to MNI space. The diagrams on the right and left sides illustrate the underlying association of face-preferential responses and RSFC.

In contrast to these results, for the FFAs, in addition to the lower number of informative clusters in the MVPA analysis, the RSFC pattern was largely divergent as exemplarily shown for two MVPA clusters informative of FFA face-selective responses (rFFA: right middle frontal gyrus extending into the precentral gyrus, lFFA: left caudate nucleus and olfactory gyrus). Only one significant common cluster was observed in the right supramarginal gyrus extending into the inferior parietal gyrus (peak: 57x − 27 < 45z; 81 voxels, p(FWE-corr.) = 0.010) using the of the right R middle frontal gyrus/precentral gyrus (rFFA) and the left caudate nucleus and olfactory gyri (− 6 6 − 15, lFFA) as seeds. The results are illustrated in Fig. 4.

### Supramodal convergence of informative RSFC patterns

The combination of RSFC correlates of individual face-preferential responses in the right and left FFA with RSFC correlates of individual voice-preferential responses in the right and left TVA can decipher supramodal convergence of RSFC patterns, i.e. combining voice- and face-preferentiality. In our case, this was evident in eight clusters (Table 5). The convergence was more prominent using right-hemispheric voice- and face-preferentiality regressors with five common clusters, whereas for the left-hemispheric regressors only one supramodal cluster was found. Two clusters derived from regressors of contralateral hemispheres.

**Figure 5:**
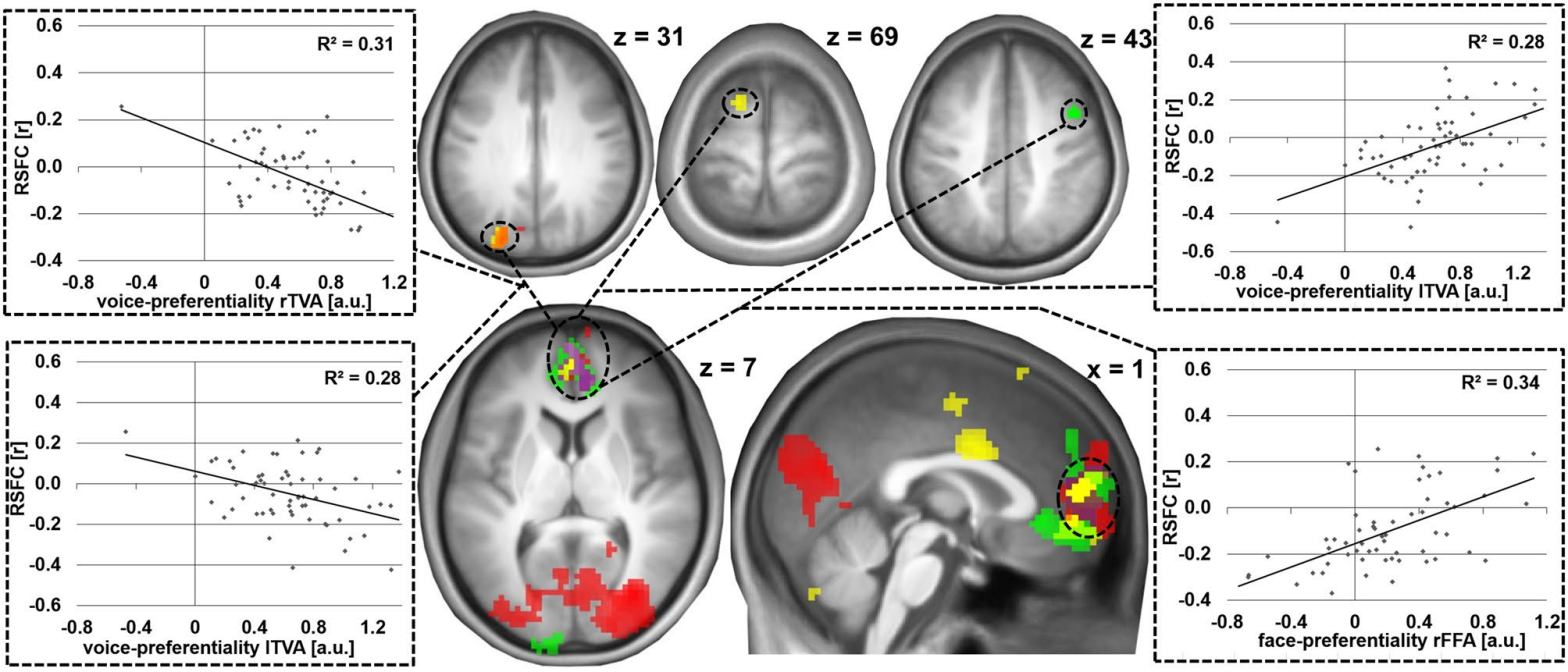
RSFC patterns informative of voice- and face-preferentiality: supramodal convergence using three regressors. Individual voice- and face-preferential responses were assessed using minimum difference criteria (for voices V > max[A, E], for faces F > max[H, O, S]). Top brain section: MVPA clusters used as seed regions [see also Fig. 2b,d] with the rTVA (red) and lTVA (yellow; overlapping region in orange) voice-preferentialities, as well as the rFFA face-preferentiality (green) as regressors. Bottom brain section: The seeds’ convergent RSFC patterns regarding individual voice-preferential responses of the right (red) and left (yellow) TVA and face-preferential responses of the right FFA (green), (purple: supramodal overlap). Results shown at a voxel-wise threshold of p < 0.001 and whole brain FWE-corrected at cluster level with a cluster threshold of p < 0.05. Coordinates refer to MNI space. The diagrams on the right illustrate the underlying association of voice-preferential responses and RSFC.

Only one region in the anterior region of the rostral mediofrontal cortex (arMFC) exhibited supramodal convergence of informative RSFC patterns for more than two regressors: Convergence of the RSFC of the rlTVA cluster in the left superior occipital gyrus with the lTVA cluster in the left frontal superior gyrus and the rFFA cluster in the right middle frontal gyrus delineated one common region in the medial frontal gyrus (including the left orbital gyrus and the anterior cingulum as well as the right and left medial frontal gyrus, peak: 0 × 54y 9z; 83 voxels, p(FWE-corr.) = 0.011) indicative of right and left TVA voice-preferentiality as well as rFFA face-preferentiality (see also Fig. 5).

## Discussion

Combining seminal experiments used to localize voice- as well as face-preferential areas in the human brain and resting state fMRI, this study provides the first description of hemodynamic functional connectivity patterns in the resting state that are associated with voice- and face-preferential cerebral responses at the primary level of the TVA and FFA.

Using functional connectivity in the resting state, we identified several clusters correlating with voice- and face-preferentiality of the TVA and FFA. For the rFFA one right frontal/precentral cluster was evident, for the lFFA two clusters, one in the left caudate/olfactory gyrus and one in the left superior temporal pole. Using the voice-preferentiality of the rTVA and lTVA as regressors, four common clusters emerged. These were widely distributed the occipital, parietal, frontal and temporal cortex. For the rTVA two additional clusters in the left occipital cortex and the right thalamus, and for the lTVA in the left frontal and right parietal cortex areas emerged. In explanatory seed-to-voxel analyses, the underlying connectivity patterns diverged markedly between the voice and face processing systems. Whereas for the TVAs a largely convergent pattern of clusters was observed, among others in the occipital gyrus and bilateral insulae, the patterns for the FFAs were mainly divergent and yielded only one common region in the right supramarginal gyrus extending into the inferior parietal gyrus.

Moreover, we identified brain areas with RSFC patterns supramodally reflecting preferential responses to both, voices and faces. One area in the anterior rostral mediofrontal cortex (arMFC) displayed a maximum of convergent RSFC patterns: its RSFC was indicative of individual voice-preferential responses of both TVAs and face-preferential responses of the right FFA.

Our results strengthen the view that cerebral voice and face processing is an evolutionary important and therefore highly preserved mechanism, which is not only evidenced by several stages of very specialized processing in the brain, starting with the regions of TVA^1–4^ and FFA^5–8^, but is also reflected in other networks, i.e. the resting state network that—per se—work independent from the aforementioned voice and face processing system. Because during resting state participants were asked to lie quiescent without specific thought. But the independence could be impaired, in case the participants would have thought of human voices and faces during the resting state measurement. To minimize this risk, we designed the experimental sequence with the resting state block first followed by the task-related parts.

The finding of a correlation of voice- and face-activation patterns with resting state parameters fits in quite well with the currently still limited literature applying both resting state and voice/face processing measurements. Previous studies found diverging regions either exclusively in the modality-specific processing areas^27,42^, both in modality specific areas and other parts of the brain^28,29^, or networks in the inferior frontal gyrus and motor regions which are not directly connected to modality specific processing^30^. It needs to be acknowledged however that a broad range of diverse data analysis techniques were used in those studies^27–30,42^ which may account for the disparities to some extent. Our comprehensive analysis on RSFC networks associated with voice- and face-preferentiality revealed large networks across whole brain, underpinning the notion that response patterns generated in basic voice and face processing modules during the perception of these cues find a reflection in the coactivation of widespread cerebral networks at rest potentially indicating processes connected to voice and face perception or a neural preparedness to respond to these stimuli. Speaking figuratively, the direct responses to stimulation with voices and faces can be imagined as the top of the iceberg, the underlying resting state network structure as the part below the surface of the sea.

It is known from the literature that resting state patterns reflect individual traits. In fact, resting state functional connectivity has been shown to be associated with behavioural tendencies, personality or states of psychiatric disease, e.g. personality traits^22,23^, moral behaviour^43^, violence proneness^25^, or the diagnosis of dementia or schizophrenia^26^. These results support the view that resting state patterns may reflect an adaptive system indicative of different brain states and function. One could speculate about the connection between basal voice and face processing systems, as assessed in our work, and higher order social functioning (e.g., emotional communication, empathy, theory of mind or moral behaviour), as effective voice and face perception appears as a prerequisite of the former to a certain degree. Certainly, however, this link remains speculative presently.

The novel and distinctive feature of this study is the combination of resting state and stimulation-based fMRI measurements for the visual and the auditory system. The resting state pattern, i.e. a stimulation-free measurement, correlates with the propensity to respond to certain stimuli. Up to now, this form of association has only scarcely been addressed. A similar approach revealed non-state-dependent cerebral markers of biased perception in social anxiety^37^. Another meta-analytic study focused on similarities in resting state functional connectivity patterns and coactivation network configurations. Using an online database activation patterns of several different tasks were pooled together. A high correlation between coactivation during task and resting-state correlation was detected^44^. In patients with first episode schizophrenia overlapping dysfunctions in the prefrontotemporal pathway were evident^45^. Our study can serve as starting point for further combined analyses of resting state connectivity and activation patterns in stimulation-based designs from a network perspective with a much more precise task design.

Convergent with previous research which provided evidence for a greater functional similarity between the hemispheres in the cerebral voice processing system than the face processing system^46–48^, in our study, both TVAs responded to voices in a sensitive (i.e. mixed contrast V > (A, E)) and preferential (i.e. minimum contrast, V > max(A, E)) manner. In contrast, in the face processing system only the right FFA responds both in a sensitive and preferential way to faces, whereas the response of the left FFA is only face-sensitive. We substantiated these results comparing voice- and face-sensitivity and -preferentiality of both hemispheres with lack of hemispheric differences in voice-preferentiality, but significant hemispheric differences in the face processing system with greater face-preferentiality in the right hemisphere. This finding is in line with previous results showing stronger and more consistent activation through faces in comparison to other stimulus categories in the right FFA compared to the left FFA^46,49^. The dominance of the right hemisphere in face-related responses is not restricted to the FFA, but is also reflected in larger activation areas to faces in the right occipitotemporal cortex and the right amygdala and an exclusive activation of the right inferior frontal gyrus^46^. Beyond this reliably replicated evidence, we found corresponding patterns in resting state measurements: The resting state patterns predicting the face- and voice-sensitivity/-preferentiality, respectively, differed showing a convergent pattern for the voice processing system and a largely divergent pattern for the face processing system as evidenced by the difference in significant overlaps of the informative connectivity patterns between the TVAs as compared to the FFAs. Thus, we conclude that the different qualities of seeing faces and hearing voices do not work analogously, but that these two systems function in a unique and distinct way, with a higher hemispheric functional similarity of the voice processing modules in comparison to the face processing system.

In our supramodal approach combining voice and face processing networks with three regressors, one common region in the medial frontal cortex correlated both voice- and face-preferentiality during resting state. The medial frontal cortex is known to be activated in higher order social cognitive processing, the anterior rostral part especially in mentalizing tasks^50^. Additionally, it is involved in complex emotion processing^13,51,52^, independent of the presentation form, e.g. visually via faces or bodies or acoustically via voices^53^. The activation of a region related to the processing of stimuli from different sensory modalities gives rise to the problem of interpreting the results. Throughout this manuscript we use the term supramodal for the locally overlapping cerebral activation by signals from different sensory modalities which can be identified using conjunction analyses, e.g. for mapping multisensory integration^41,54^. Limitations of the technique are that in our case the common region constitutes only a small part in comparison to the complete connectivity pattern from each source, and that the local overlap not mandatorily represents a direct interaction or integration of signals from both sources, but might indicate that the overlap region is simply linked to processing information from several sensory modalities.

While the medial frontal cortex is not consistently activated in stimulation experiments designed to localize voice- or face-specific brain areas, this notion would still appear quite plausible as effective processing of voices and faces might well be required as basis for a variety of higher order social communication functions. In line with this, frontal areas were involved in the processing of incongruent but not congruent audiovisual emotional stimuli^55,56^ and revealed emotion-specific activation regardless of the sensory modality of the emotional cue^53^. Whereas many studies assessing higher order social processing employed emotional stimuli^13,51,52^, it is quite notable, that we found a convergence in this region even based on experimental designs without explicit emotional connotations. Limitations concerning the assessment of neutral vs. emotional stimuli are discussed below.

This seems to corroborate the notion that higher order social cognitive processes are linked to basic voice and face perception irrespective of emotional information communicated via these stimuli. On the other hand, one might argue that there is no such thing as a voice or a face completely devoid of emotional information in two ways: First, also stimuli not intended to carry emotional information by their sender may well contain subliminal emotional cues and, second, even a putative completely neutral voice or face may automatically be scanned for emotional information and therefore become linked to emotion processing irrespective of its lack of emotional cues. Previous results hint for a variability in the emotional perception of voices and faces depending on the previously experienced sensory input^57,58^.

The posterior superior temporal sulcus (pSTS), which has been shown to integrate simultaneously presented auditory and visual stimuli^10^, did not show an overlap of connectivity patterns indicating both voice- and face-preferential responses. So, the pSTS’s role in combined face and voice processing might be more closely linked to the sensory integration of these stimuli during their simultaneous perception and thus not be detectable in the resting state.

Our work builds on the manifold confirmed and pioneering findings of regions that are preferentially activated by human stimuli in comparison to environmental cues, i.e. especially the voice-preferential activation of the TVA and the face-preferential activation of the FFA^4–6^. And it broadens the perspective from specialized regions for different tasks to a network perspective of regions exhibiting preferential responses both during and in the absence of human nonverbal cues. One could speculate that the relevance of this finding lies in the reflection of relevant social situations during resting state, possibly including imagination of nonverbal cues. But to corroborate these ideas, further research is necessary.

The unique quality of our data stems from the combination of these individual cerebral processing characteristics of social stimuli with resting state functional connectivity maps in a relatively large cohort. And it adds to the growing number of findings which advocate a readjustment of our view from specialized regions in the brain responding to certain stimuli to a larger network perspective involving a multitude of regions across the whole brain in the presence and absence of tasks or/and stimuli. The specificity of the activation is mediated not by the activation of single specialized regions itself but by the combination of simultaneously activated networks and therefore strengthens the view of a network perspective.

As gender-specific connectivity patterns were observed e.g. in the correlation of RSFC with personality traits^22^, this aspect represents a limitation of the present study which focused on gender- and age-independent connectivity patterns. Due to the limited sample size, we did not perform subgroup analyses. Moreover, we did not assess and therefore were able to correct for personality trait measures, such as the five-factor model of personality, which was shown to be associated with RSFC patterns^22^ and might therefore also represent a moderator of the RSFC patterns associated with the propensity to respond to human voices and faces.

Although based on seminal standard experiments to assess voice- and face-sensitive and -preferential responses enabling direct comparisons with many previous studies, certain design-specific factors may have influenced the outcome of our study and should therefore be addressed in further research: For one, the task-set differed considerably between the voice and face processing experiments (passive listening vs. one-back task) with potential influence on the attentional status. As a further limitation we would like to address the problem of the assessment of human stimuli as neutral vs. emotional. Though not included in the experiments as explicit factor employing face pictures with predominantly neutral expression, low-level emotional information in the experimental stimuli may have impacted the RSFC patterns predictive of cerebral voice- and face-preferentiality.

As a conclusion, these results emphasize that the individual cerebral propensity to respond to human voices and faces is reflected in the brain’s activation patterns also in the absence of these cues as a possible neural corelate of mental reflections on relevant social situations including imagination of nonverbal cues during “resting” state. The stronger convergence of informative connectivity patterns for the TVAs’ cue selectivity in contrast to the FFAs’ may indicate a higher hemispheric functional similarity of the voice processing modules. The supramodal convergence of such informative connectivity patterns, in turn, points to the anterior medial prefrontal cortex as shared neural resource in supramodal voice and face processing or potentially nonverbal communication. Similar to the underwater perspective on an iceberg, this experimental approach may open up interesting avenues to the investigation of voice and face processing. In this regard, the resting state connectivity patterns correlating with individual voice and face selectivity may aid the understanding of cerebral voice and face preference from a network perspective.

## Supplementary Information

Supplementary Figure 1.

## Data Availability

The datasets generated during and/or analysed during the current study are available from the corresponding author on reasonable request.
