# Supplementary material for: Correlates of individual voice and face preferential responses during resting state
Source: Sci Rep. 2022 May 3;12:7117. doi: 10.1038/s41598-022-11367-6 (PMC9065073; doi:10.1038/s41598-022-11367-6)
Supplement: Supplementary file 1 — Supplementary Figure 1. [file 41598_2022_11367_MOESM1_ESM.pdf]

Correlates of individual voice and face preferential responses during resting state

Kathrin N. Eckstein, Dirk Wildgruber, Thomas Ethofer, Carolin Brück, Heike Jacob, Michael Erb, and Benjamin Kreifelts

Supplemental Material

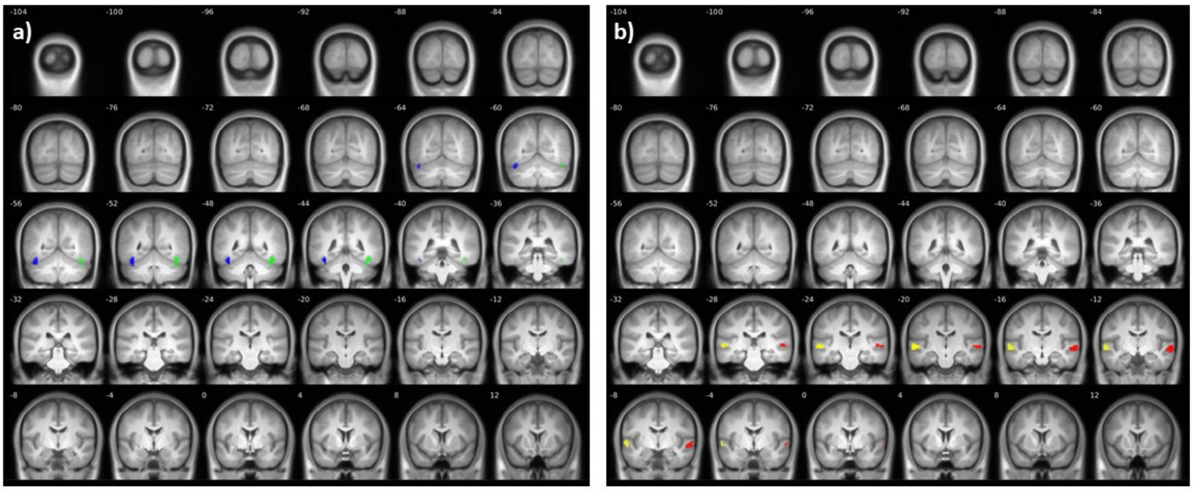

Supplemental Figure 1. Whole brain slices of the ROIs FFA and TVA.
Axial whole brain slices are given a) for the fusiform face area (rFFA in green, lFFA in blue), and b) the temporal voice area (rTVA in red, lTVA in yellow), rendered onto the mean anatomical scan of the study population. The functional ROIs (i.e. FFA and TVA) were identified picking the maximum activation in the fusiform gyrus and in the temporal lobe respectively, and defining the surrounding 100 most sensitive voxels as masks.
